# Optical Properties and Defects of Single-Crystal LiNbO_3_:Tb^3+^ Plates with a Post-Threshold Concentration of Terbium Ions

**DOI:** 10.3390/ma19184021

**Published:** 2026-09-21

**Authors:** Nikolay V. Sidorov, Mikhail N. Palatnikov, Alexander V. Skrabatun, Lyubov A. Bobreva, Roman A. Titov, Olga V. Palatnikova, Oleg A. Klimenko, Alexander Yu. Pyatyshev

**Affiliations:** 1 Tananaev Institute of Chemistry—Subdivision of the Federal Research Centre, Kola Science Centre of the Russian Academy of Sciences (ICT RAS), 184209 Apatity, Russia; n.sidorov@ksc.ru (N.V.S.); m.palatnikov@ksc.ru (M.N.P.); l.bobreva@ksc.ru (L.A.B.); r.titov@ksc.ru (R.A.T.); o.palatnikova@ksc.ru (O.V.P.); 2 P. N. Lebedev Physical Institute of the Russian Academy of Sciences, 119991 Moscow, Russia; skrabatunav@lebedev.ru (A.V.S.); klimenkooa@lebedev.ru (O.A.K.); 3 Department of Physics and Technical Mechanics, MIREA—Russian Technological University, 119454 Moscow, Russia; 4 Skolkovo Institute of Science and Technology, Bolshoy Boulevard 30, Bld. 1, 121205 Moscow, Russia

**Keywords:** lithium niobate, terbium, absorption, Raman scattering, IR absorption spectra, OH^−^-group

## Abstract

The macro- and microstructures of LiNbO_3_:Tb^3+^(2.25 wt%) and LiNbO_3_:Tb^3+^(2.9 wt%) crystals were studied. It was shown that a slight increase in terbium content dramatically alters the growth domain structure and enhances fractal self-organization. The defect structure features of LiNbO_3_:Tb^3+^(2.25 wt%) and LiNbO_3_:Tb^3+^(2.9 wt%) single-crystal plates with an after-threshold terbium concentration were studied using optical absorption spectra, Raman scattering spectra in the frequency range of 65–4000 cm^−1^, and IR absorption spectra in the frequency range of 1000–8000 cm^−1^. It was shown that the LiNbO_3_:Tb^3+^(2.25 wt%) plate has a lower concentration of Nb_Li_ point defects and a higher stoichiometry compared to the LiNbO_3_:Tb^3+^(2.9 wt%) plate. A good match was found between the Raman spectra of the LiNbO_3_:Tb^3+^(2.25 wt%) and LiNbO_3_:Tb^3+^(2.9 wt%) plates throughout the entire studied range. A significant difference was found in the frequencies of the lines corresponding to stretching vibrations of OH^−^-groups in the vibrational spectra of the studied plates. This observation is explained by the fact that OH^−^-groups in the LiNbO_3_ crystal structure are defects, and, therefore, the stretching vibrations of OH^−^-groups are not fundamental lattice vibrations. The existing selection rules for fundamental lattice vibrations, developed on the basis of group theory, do not apply to the defect sublattice.

## 1. Introduction

The Tb^3+^ ion exhibits favorable luminescent properties arising from 4f–4f or 5d–4f transitions [1]. These properties can facilitate the development of materials for optoelectronic devices based on isomorphous nonlinear optical crystals of lithium niobate (LiNbO_3_) and lithium tantalate (LiTaO_3_). Terbium-doped LiNbO_3_ and LiTaO_3_ crystals exhibit the effect of a photoinduced variation in the refractive index (photorefraction effect or optical damage) and intense luminescence in the range of 400–600 nm, as well as a long afterglow [2,3,4,5,6,7,8,9,10,11,12,13]. In [7], during a study of the luminescent properties of the LiNbO_3_:Tb^3+^ crystal, the possibility of creating an active, nonlinear laser element emitting in the blue region of the spectrum was demonstrated. The aforementioned applications of the LiNbO_3_:Tb^3+^ crystals require the active medium to be highly structurally homogeneous and contain minimal structural defects. It is also worth noting that LiNbO_3_ has no absorption in the range of ≈250–5300 nm, which allows it to be used to create materials that operate within the specified wavelength range.

LiNbO_3_:Tb^3+^ crystals of any doping level have a complex defect structure, which imposes limitations on their application. This is due to the fact that the lithium niobate matrix is an oxygen-octahedral phase of variable composition with a wide homogeneity region in the phase diagram [14]. In this case, the maxima on the liquidus and solidus curves are strongly smoothed, and the position of the dystectic point does not coincide with the stoichiometric composition. This indicates partial dissociation of the compound in the solid and liquid states. Such crystalline structures have a very diverse and very high defect density: point and volume (microstructure) stoichiometry defects, structural and concentration heterogeneity of the composition throughout the volume of the crystal, etc. [15,16,17,18,19,20,21,22,23]. The features of the phase diagram [14] and the oxygen-octahedral structure of the LiNbO_3_ matrix—in which only two-thirds of the oxygen octahedra O_6_ are filled with the main ions Li^+^, Nb^5+^ and impurity metals (Me) and a third of the octahedra remain empty [24,25]—make it possible to effectively regulate the state of defects in the LiNbO_3_:Tb^3+^ crystal over a wide range by changing the concentration of the Tb^3+^ ion, changing the stoichiometry of the matrix (the value of R = [Li]/[Nb]), and changing the technology for growing the crystal [15].

Previously [15,20,21,26,27], the presence of a concentration threshold (2.2–2.3 wt% Tb^3+^ ions) was established, upon crossing which the defectiveness increases and the structural perfection and compositional homogeneity of LiNbO_3_:Tb^3+^ crystals deteriorate, which is manifested, among other things, in a decrease in the Curie temperature and an increase in the photorefraction effect. The decrease in the Curie temperature is explained [15,20,26,27] by an increase in the concentration of point defects V_Li_—lithium octahedra LiO_6_, in which the Li^+^ ion is absent. Moreover, the magnitude of the coercive field in after-threshold LiNbO_3_:Tb^3+^ crystals is less than that in under-threshold crystals [15]. This fact is important for the development of laser radiation converters based on after-threshold LiNbO_3_:Tb^3+^ (≥2.2 wt%) crystals, which consist of functional elements that are periodically polarized, submicron-sized domains with flat boundaries formed on oriented single-crystal plates [28,29,30,31]. In this case, the fabrication of these elements requires LiNbO_3_:Tb^3+^ plates of high optical quality and structural perfection with a low coercive field.

An important feature of a LiNbO_3_ crystal of any composition grown in an air atmosphere is the presence of bulk defects in the form of OH^−^-groups and associated complex defects such as (Nb_Li_^4+^)-OH, (V_Li_^−^)-OH, etc. [18,19,32,33,34,35,36]. To date, the localization features of complex defects such as (V_Li_^−^)-OH, (Nb_Li_^4+^)-OH, (Me_Li_)-OH (Me is an impurity metal), etc., in the LiNbO_3_:Tb^3+^ crystal structure and their influence on the optical properties of the crystal have been studied little in the literature. However, such defects can have a noticeable effect on the optical quality of functional materials for converting laser radiation based on a LiNbO_3_:Tb^3+^ crystal, the local structure of the material, and the formation of microstructures in the material. It should be noted that the presence of defects in the form of OH^−^-groups in LiNbO_3_ crystals of any composition also has a noticeable effect on practically significant properties of the crystal such as conductivity, photorefraction, and hologram fixation [15,19,24,25,37,38,39,40,41,42].

In the present work, optical and vibrational spectroscopy were used to investigate the optical homogeneity and defect state features of LiNbO_3_:Tb^3+^(2.25 wt%) and LiNbO_3_:Tb^3+^(2.9 wt%) single-crystal plates with an after-threshold concentration of terbium ions. These concentrations were chosen because 2.25 wt% lies within the concentration threshold of 2.2–2.3 wt%, where the most significant changes in properties are expected, and 2.9 wt% allows for the further evolution of the defect structure to be observed with increasing Tb^3+^ content. This will allow us to observe how changes in structure and composition affect the optical properties.

It is widely known that the stretching vibrations of OH^−^-groups cause extremely small changes in the polarizability of the LiNbO_3_ crystal unit cell, and, therefore, the corresponding lines in the Raman spectrum have very low intensity. For this reason, there are virtually no studies in the literature on the Raman spectra of complex defects caused by the presence of O-H bonds in the structure of LiNbO_3_ crystals of any composition. At the same time, OH^−^-groups absorb IR radiation very strongly, and, therefore, IR absorption spectroscopy is a much more sensitive method of studying the various types of defects caused by the presence of O-H bonds in the structure of the LiNbO_3_ crystal than Raman spectroscopy [18,19,32,33,34,35,36].

## 2. Materials and Methods

LiNbO_3_:Tb^3+^(2.25 and 2.9 wt%) single crystals were grown by the Czochralski method in an air atmosphere in platinum crucibles with a diameter of 75 mm in the direction of the polar axis (z-cut) at constant rotation (16 rpm) and translation (0.8 mm/h) rates. The crystal increment rate was 1.08 mm/h. A granulated batch of congruent composition ([Li_2_O] = 48.6 mol.%) with a high bulk density (~3.1 g/cm^3^) was used [43]. The batch was synthesized using niobium pentoxide (Nb_2_O_5_—Solikamsk Magnesium Plant (Solikamsk, Russia), in compliance with Russian technical standard No. 1763–025-00545484–2000; impurity content did not exceed 1 × 10^−4^ wt%) and lithium carbonate (Li_2_CO_3_—impurity level less than 3 × 10^−4^ wt%). LiNbO_3_:Tb^3+^ crystals were grown in a Kristall 2 growth facility (Voroshilovgradsky zavod electronnogo mashinostroeniya, Voroshilovgrad, USSR) equipped with an automatic crystal diameter control system. Terbium was introduced into the batch in the form of Tb_2_O_3_ (99.99% purity). The process of single-domain formation of LiNbO_3_:Tb^3+^(2.25 and 2.9 wt%) crystals was carried out at high temperatures (~1238–900 °C) by electrodiffusion annealing in a Lantan setup (Voroshilovgradsky zavod electronnogo mashinostroeniya, Voroshilovgrad, USSR). The degree of single-domain formation of the grown single crystals was controlled by a piezoacoustic method. The preparation of LiNbO_3_:Tb^3+^ single crystals is described in more detail in [15].

**Figure 1:**
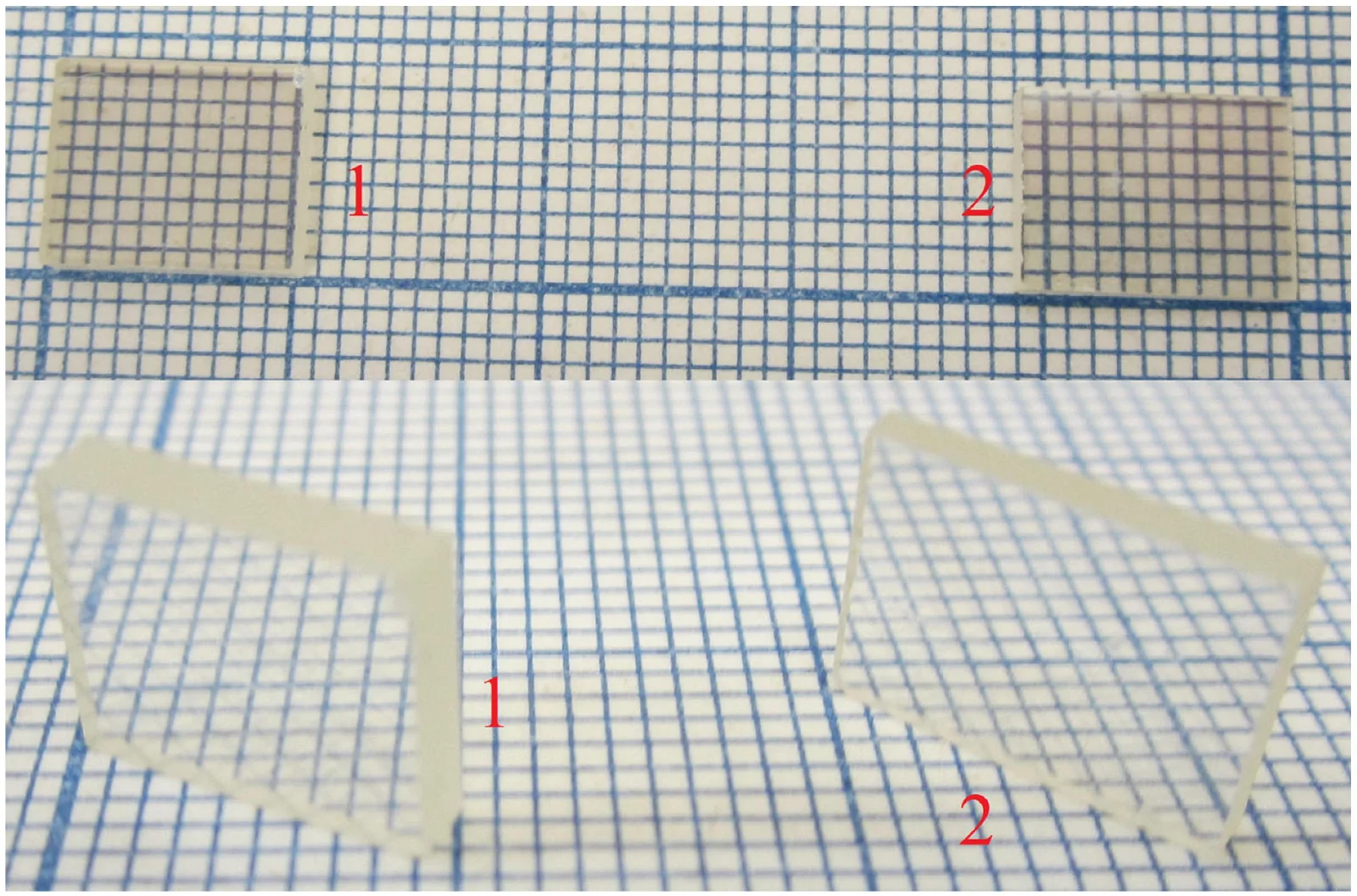
Photographs of Z-oriented crystal plates: LiNbO_3_:Tb^3+^(2.25 wt%)—1; LiNbO_3_:Tb^3+^(2.9 wt%)—2.

The samples for the studies were cut from the central parts of single-crystal boules to exclude the influence of a possible impurity concentration gradient along the growth axis and to ensure comparability of the results. The Z-oriented plates had dimensions of 8.30 × 10.11 × 2.27 mm^3^ for LiNbO_3_:Tb^3+^(2.25 wt%) and 8.09 × 11.43 × 1.55 mm^3^ for LiNbO_3_:Tb^3+^(2.9 wt%) (see Figure 1). Initially, the single-crystal plates were ground using an aqueous suspension of diamond powder with a grain size of ~8 μm. Additional grinding of the samples was carried out using an aqueous suspension of diamond powder with a grain size of ~3 μm. After each grinding stage, the surface of the single-crystal plates was washed successively with volatile liquids: a mixture of ethyl alcohol and hexane and then trichloroethylene. Single-crystal plates were polished using chemical mechanical polishing with a Magnum-Top-Duo diamond suspension (1 μm diameter single-crystal diamonds, MetCata, Kaufering, Germany) on a MetaServ 3000 Model 95-2940 grinding and polishing machine (Buehler, China). After polishing, the surface of the single-crystal plates was also successively rinsed with a mixture of ethyl alcohol and hexane and then with trichloroethylene.

Crystal microstructure was studied using a Thixomet image analyzer, which consists of an Axio Observer.D1m (Carl Zeiss, Oberkochen, Germany) microscope coupled to a video camera. Brightfield mode and differential interference contrast (DIC) were used for this study.

Optical (190–1100 nm range) and IR absorption spectra of the plates were recorded on a UNICO 2800 UV/VIS spectrophotometer (United Products & Instruments, NJ, USA) and a Bruker VERTEX 70v FTIR spectrometer (Bruker, Karlsruhe, Germany), respectively. IR absorption spectra were recorded at a pressure of less than 1 hPa. Raman spectra were recorded using a BWS465-532S i-Raman Plus spectrometer (B&W Tek, Plainsboro Township, NJ, USA).

## 3. Results

### 3.1. Study of Macro- and Microstructure

Various microscopic and macroscopic parameters of LiNbO_3_:Tb^3+^ crystals, such as the impurity distribution coefficient K_D_(Tb), the Curie temperature, features of the optical characteristics, and the parameters and volume of the unit cell, vary significantly over a wide range of dopant concentrations [21,26,27].

Growing heavily doped LiNbO_3_:Tb^3+^ crystals that possess a relatively uniform dopant distribution throughout the grown crystal from a melt using the Czochralski method presents a complex technological challenge. Axial and radial temperature gradients in the melt, combined with the ratio of the crucible diameter to the growing crystal diameter and its rotation speed, determine the relationship between the hydrodynamic flows in the crucible, which influence the thickness of the diffusion layer and the shape of the crystallization front and, consequently, the defect macro- and microstructure.

Crystallization from a melt occurs through the addition of ionic clusters to the growing crystal, the structure and composition of which directly depend on the melt composition and, consequently, on the concentration of the alloying component [44,45,46]. Accordingly, during crystallization from melts with different compositions, all other things being equal, the structure and composition of the ionic complexes will differ slightly. This can lead to variations in the defect structure and macro- and microstructure of LiNbO_3_:Tb^3+^ crystals with different impurity concentrations.

In the growth and study of LiNbO_3_:Tb^3+^ crystals—where the dopant distribution coefficient depends on its concentration—data on crystal growth mechanisms as a function of impurity concentration are of great importance for improving growth technology [47].

Moreover, the melt composition near the crystallization front during LiNbO_3_:Tb^3+^ crystal growth can change due to the impurity distribution coefficients K_D_(Tb) deviating from unity, which can lead to increased crystal defects. To minimize such effects, a special thermal assembly design is typically employed, ensuring a virtually uniform temperature across the entire crystallization front [26].

Optical microscopy was applied to analyze the macro- and microstructure of LiNbO_3_:Tb^3+^ crystals of varying compositions. This allowed us to assess the influence of impurity concentration on the morphology and structural characteristics of the growth macro- and microdefect structure of LiNbO_3_:Tb^3+^ crystals and, consequently, on the crystal growth mechanisms.

Z-cut samples were analyzed, as the defect structure of nonpolar-cut LiNbO_3_:Tb^3+^ crystals is similar to the periodic defect structure of all rare earth-doped lithium niobate crystals and has been described in detail in [21,27,48,49].

**Figure 2:**
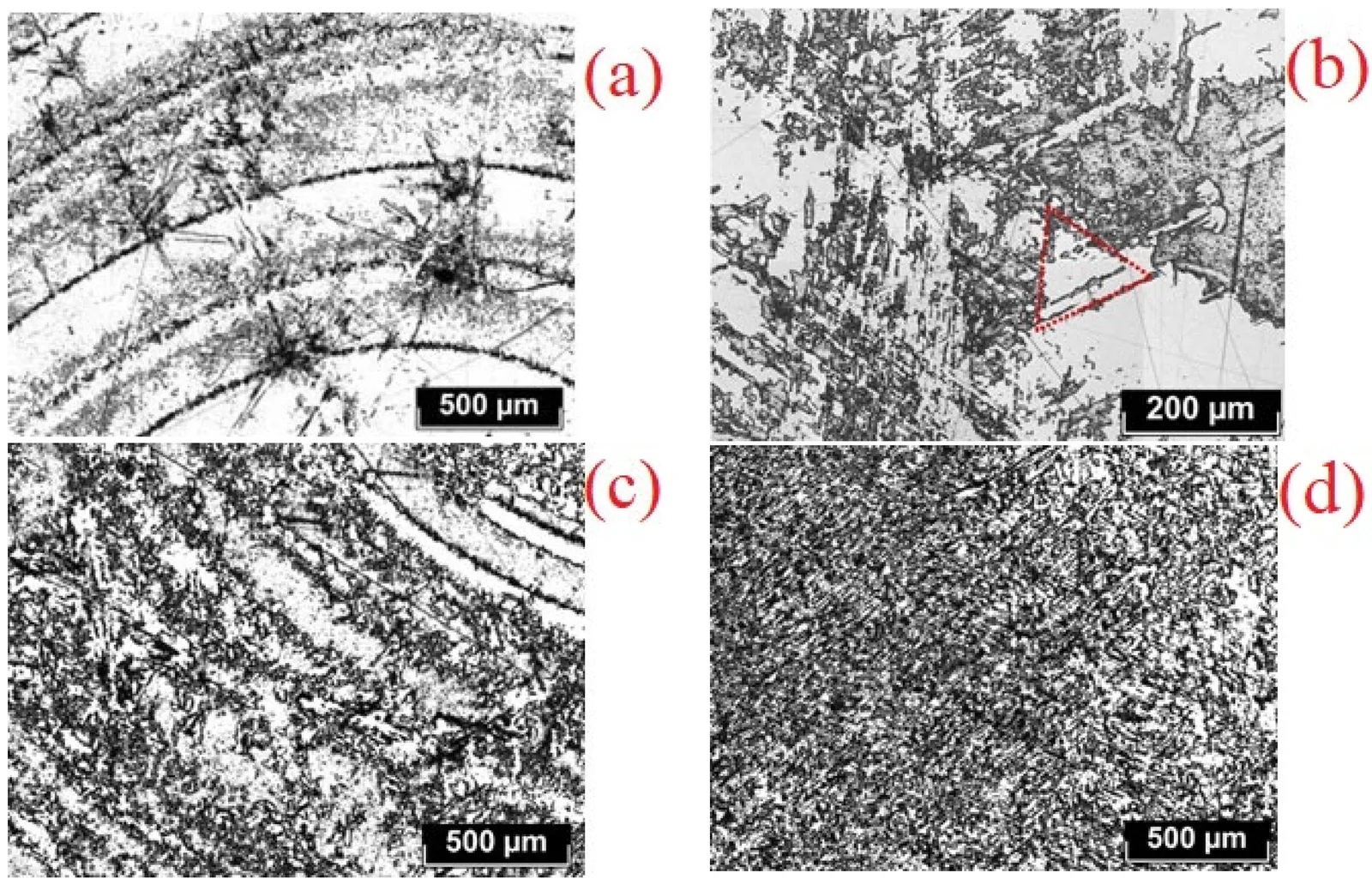
Macro- and microstructure of LiNbO_3_:Tb^3+^(2.25 wt%) (**a**,**b**) and LiNbO_3_:Tb^3+^(2.9 wt%) (**c**,**d**) crystals. Z-cut.

Figure 2 shows images of the Z-surface of LiNbO_3_:Tb^3+^ crystalline plates of varying compositions. When studying polar Z-cut LiNbO_3_ crystals, the mutually opposite crystallographic orientation of domains of opposite signs makes it possible to observe a fairly distinct pattern of the macrodomain structure using the etching method (Figure 2a).

For the LiNbO_3_:Tb^3+^(2.25 wt%) crystal, the mutual orientation of concentric macrodomains of various signs (growth bands) on the Z-plane of the crystal, perpendicular to the growth axis, is typical of lithium niobate crystals doped with rare earth elements (REEs) [26,48,49] (Figure 2a). Here, as for all LiNbO_3_ crystals doped with REEs, the macrodomain structure of the Z-cut of LiNbO_3_:Tb^3+^ crystals follows the shape of the isotherm at the phase boundary, and the specific arrangement of the macrodomains (concentric growth bands) is determined by convection–diffusion processes in the melt. Moreover, for the LiNbO_3_:Tb^3+^(2.25 wt%) crystal, the macrodomain structure, as a whole, approaches the shape of regular concentric rings, which indicates a relatively stable and specifically ordered convection in the melt (Figure 2a). At the same time, the macrostructure of this crystal consists of growth domain rings of varying width and texture (Figure 2a). Against the background of the growth macrostructure, there are inhomogeneities that, at a relatively high terbium concentration, form the centers of the second phase formation located inside the growth rings (Figure 2a). A slight increase in the terbium concentration leads to a significant evolution of the macrostructure of the LiNbO_3_:Tb^3+^ crystal. For the LiNbO_3_:Tb^3+^(2.9 wt%) crystal, the growth domain rings begin to merge, and only individual weakly expressed elements of the organized macrostructure are observed (Figure 2c). Moreover, in some places, a continuous etching field of the negative domain is observed (Figure 2d). Thus, a slight increase in the terbium concentration leads to a significant evolution of the macrostructure of the LiNbO_3_:Tb^3+^ crystal. With an increase in the terbium concentration of less than 1 wt%, almost no growth rings were formed. Something similar was observed for post-threshold LiNbO_3_:Er crystals at high impurity concentrations, when growth bands on the Z-cut of the crystal were completely absent [50].

Concentric macrodomains of different polarities (growth bands) in LiNbO_3_:REE crystals arise under non-stationary growth conditions due to periodic temperature oscillations near the crystal–melt interface caused by convection currents in the melt and changes in the growth rate in the boundary layer, as well as the potential participation of ionic complexes of different compositions and structures in the crystallization process. These processes are apparently caused by a discrepancy between the rate of convection–diffusion transport of various ionic complexes in the melt containing a rare earth cation to the crystallization front and the crystallization rate itself. Clearly, such a mechanism will operate within a specific impurity (REE) concentration range. This confirms the significant difference in the composition and structure of ionic complexes in the melt during the growth of LiNbO_3_:Tb^3+^(2.25 wt%) and LiNbO_3_:Tb^3+^(2.9 wt%) crystals, given that the crystal growth parameters (temperature gradient, pulling rate, and crystal rotation rate) differ only slightly.

Due to the large number of ionic complex types and complex diffusion–convection processes in the melt, crystallization during the growth of post-threshold LiNbO_3_:Tb^3+^ crystals is significantly nonequilibrium. In this case, studying the growth structure allows us to base the description of the results on the ability of the melt–crystal system to self-organize. This is facilitated by a flow of thermal energy from an external source and dissipated by the system, which enables the system to autonomously form structures [51].

Moreover, pronounced self-organization processes are observed in the LiNbO_3_:Tb^3+^(2.25 wt%) crystal (Figure 2b). Thus, seemingly chaotically located elements of the microdomain structure form fractal structures in the LiNbO_3_:Tb^3+^(2.25 wt%) crystal, on the one hand, reflecting the symmetry of the crystal, and, on the other hand, close in shape to the Sierpinski triangle (Figure 2b). One of the triangles is highlighted in Figure 2b by the red dotted line. This type of self-organization in the form of fractal Sierpinski triangles was most clearly observed in [49] on the Z-cut surface of the LiNbO_3_:Cu:Gd crystal. A much weaker manifestation of self-organization of this type is observed in the LiNbO_3_:Tb^3+^(2.25 wt%) crystal (Figure 2b).

Moreover, in the LiNbO_3_:Tb^3+^(2.9 wt%) crystal, self-organization elements spread across a significant portion of the crystal’s volume. A characteristic region of such a fractal structure is shown in Figure 2d.

### 3.2. Optical Absorption Spectra

In the most perfect structure of the LiNbO_3_ crystal of a strictly stoichiometric composition (R = 1), the presence of defects in the form of OH^−^-groups is impossible. Because real lithium niobate crystals are grown by the Czochralski method in an air atmosphere, they necessarily contain defects in the form of OH^−^-groups [18,19,32,33,34,35,36]. Hydrogen in the unit cell inevitably binds with oxygen, forming complex defects: (V_Li_^−^)-OH, (Nb_Li_^4+^)-OH, (Me_Li_)-OH (Me is a metal impurity), etc. According to X-ray structural analysis data [15,20,21,26,27], with a modification in the R value and the concentration of the Tb^3+^ dopant in the LiNbO_3_:Tb^3+^ crystal, the lengths of the Nb-O, Li-O, Tb-O, Nb-Nb, Nb-Li, Nb-Tb, and Li-Tb bonds change noticeably. In this situation, the shape of the oxygen-octahedral clusters of the LiO_6_, NbO_6_ and MeO_6_ structure (Me is an impurity metal or a V vacancy) and the arrangement of the hydrogen atoms bound in the crystal structure with oxygen by hydrogen bonds change accordingly, which leads to a variation in the electro-optical, optical, and nonlinear optical properties of the crystal [15,19,26,27]. The abovementioned effects of the disordering of the structure can be detected by analyzing the optical absorption spectra, especially the fundamental absorption edge of the LiNbO_3_:Tb^3+^ crystal, as well as the spectra of the fundamental vibrations of the crystal lattice, which are very sensitive to changes in atom–atom interactions in the crystal when the bond lengths between atoms change.

**Figure 3:**
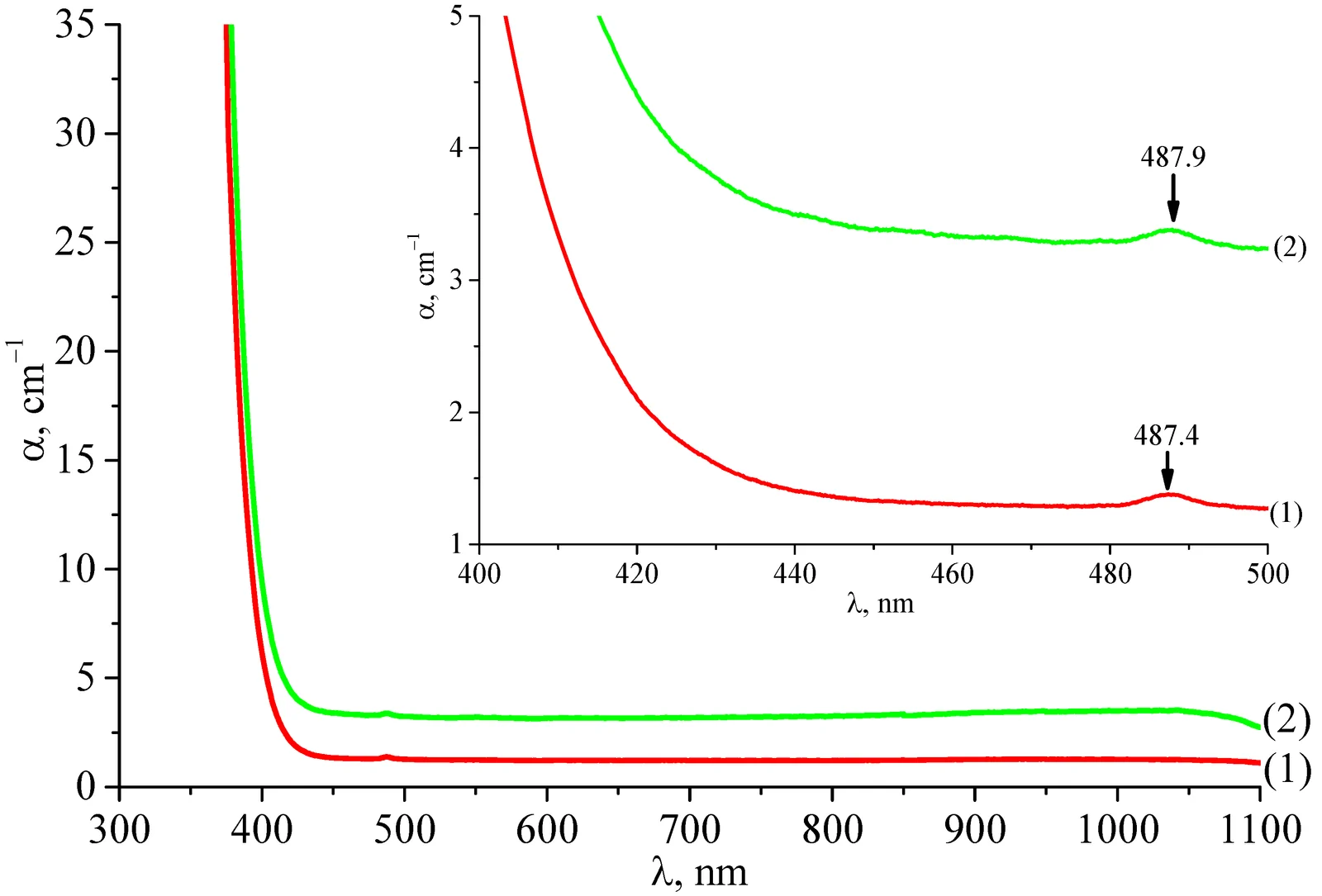
Optical absorption spectra of LiNbO_3_:Tb^3+^(2.25 wt%) (curve 1) and LiNbO_3_:Tb^3+^(2.9 wt%) (curve 2) plates in the visible and near-IR ranges. The inset shows an enlarged view of the change in the spectral position of the absorption maximum of the terbium ion.

The optical absorption spectra of LiNbO_3_:Tb^3+^(2.25 wt%) and LiNbO_3_:Tb^3+^(2.9 wt%) plates, recorded in the visible and near-IR ranges, can be seen in Figure 3.

Based on the data in [52], the absorption band near 487 nm can be attributed to the ^7^F_6_ → ^5^D_4_ transition of the Tb^3+^ ion in the LiNbO_3_ crystal. It is evident from Figure 3 that with increasing terbium concentration, the line corresponding to this transition experiences a slight shift to the long-wavelength region. To assess the structural perfection and compositional homogeneity of the crystal, important information can be obtained by analyzing the features of the fundamental absorption edge. The position of the fundamental optical absorption edge of LiNbO_3_ crystals of different compositions is mainly determined by the energy of the transition of valence electrons from the 2p orbitals of the O^2−^ atoms to the 4d orbitals of the Nb^5+^ atoms. Thus, the valence electron state of the O^2−^ oxygen atoms directly affects the position of the absorption edge of the LiNbO_3_ crystal. The interaction of positive and negative ions in the crystal leads to their polarization and a change in the electron cloud. If the dopant ions enhance the polarization of oxygen ions O^2−^, the change in the electron cloud also increases. As a result, the energy required for the electron transition decreases. This leads to a shift of the absorption edge toward the long-wavelength region of the spectrum. With a decrease in the polarization of oxygen ions O^2−^ by dopant ions, the absorption edge shifts toward shorter wavelengths. The ability to create such polarization depends on the polarizing ability of the ion, which is characterized by the ionic potential Φ = Z^2^/r, where Z and r are the effective charge and radius of the ion, respectively. In [53], the values of the ionic potential Φ decrease in the series for Nb^5+^, Tb^3+^, and Li^+^ ions and are 52.5, 20.51, and 4.2, respectively. Because the polarizing ability of the Tb^3+^ ion is significantly higher than that of the Li^+^ ion, the localization of Tb^3+^ ions in lithium positions leads to a shift of the fundamental absorption edge toward the long-wavelength region of the spectrum.

Establishing the localization of the rare earth ion Tb^3+^ in the LiNbO_3_ crystalline matrix also allows us to understand how the doping element affects the defect sublattice and, as a consequence, the general properties of the crystal. The introduction of rare earth ions into the crystal structure of LiNbO_3_ has common features. In [15,20,53,54,55], it was shown that in the crystal structure of LiNbO_3_, there are several octahedral positions that can be occupied by a rare earth dopant: the main positions of Li and Nb and a free octahedron. The data presented in [55,56] showed that rare earth ions are predominantly located in the positions of the Li ion, but, due to their large size, they are displaced from the center of the O_6_ oxygen octahedron to the nearest oxygen plane by ≈0.45 Å. In [57], it is believed that rare earth ions in the structure of the LiNbO_3_ crystal also occupy the main positions of Nb^5+^ ions (point defect Tb_Nb_^2−^), forcing Nb^5+^ ions to be introduced into vacant octahedra with the formation of a point defect Nb_V_, thereby creating clustering in the cation sublattice, which increases the disordering of cations and vacancies along the polar axis.

In this paper, we investigated LiNbO_3_:Tb^3+^ crystals grown from a doped congruent LiNbO_3_ melt. It should be noted that doping changes the crystal stoichiometry (the R value). The congruent LiNbO_3_ crystal (R = 0.946) is lithium-deficient. The compensating V_Li_^−^ defects present in the congruent crystal, which ensure the electroneutrality of the crystal, are the result of the lithium deficiency. Nb_Li_^4+^ defects are formed primarily as a result of an excess of niobium, which occupies the main lithium positions in the LiNbO_3_ crystal lattice. Furthermore, along with V_Li_^−^ and Nb_Li_^4+^ point defects, defects in the form of hydrogen atoms bonded to oxygen atoms by hydrogen bonds (OH^−^-groups) are formed in the crystal lattice during crystal growth from air. The point defect center (V_Li_^−^) attracts a hydrogen atom, which leads to the formation of a complex defect (V_Li_^−^)-OH^-^.

Using the X-ray structural analysis method and the method based on the filling factors of the G(Li, Nb, Nb_Li_, and Nb_V_) positions, given in [26], we calculated the stoichiometry value (R) of LiNbO_3_:Tb^3+^ crystals. The results showed that R = 1.005 for the LiNbO_3_:Tb^3+^(2.25 wt%) crystal and R = 0.965 for the LiNbO_3_:Tb^3+^(2.9 wt%) crystal (Table 1). It is believed that the closer the value of R is to unity, the shorter the wavelength of the fundamental absorption edge and the smaller the number of Nb_Li_ point defects in the crystal lattice. According to X-ray structural analysis data [26], in LiNbO_3_:Tb^3+^(2.25 wt%) and LiNbO_3_:Tb^3+^(2.9 wt%) crystals, there are practically no point defect centers (Nb_Li_^4+^), and, accordingly, there is a minimal number of point defects (V_Li_^−^). From Figure 3, it follows that for the LiNbO_3_:Tb^3+^(2.25 wt%) plate, there is a smaller shift of the fundamental absorption edge toward shorter wavelengths (λ_20_ = 385.55 nm) compared to the plate with LiNbO_3_:Tb^3+^(2.9 wt%) (λ_20_ = 387.1 nm) (Figure 3 and Table 1). This fact indicates a lower quantity of point defects (Nb_Li_^4+^) and, accordingly, complex defects (V_Li_^−^)-OH^-^ in LiNbO_3_:Tb^3+^(2.25 wt%) compared to LiNbO_3_:Tb^3+^(2.9 wt%).

A concentration shift of the absorption lines of chromium ions in a yttrium–aluminum garnet crystal was previously observed [58]. The authors attributed the observed shift to the expansion of the crystal lattice. A change in the spectral position of the absorption bands with increasing chromium ion concentration was also observed for LiNbO_3_:Cr^3+^ crystals [59]. According to X-ray structural analysis data [20,26,27], an increase in the concentration of terbium ions leads to a change in the parameters of the crystal lattice and the placement sites.

### 3.3. Raman Scattering Spectra

**Figure 4:**
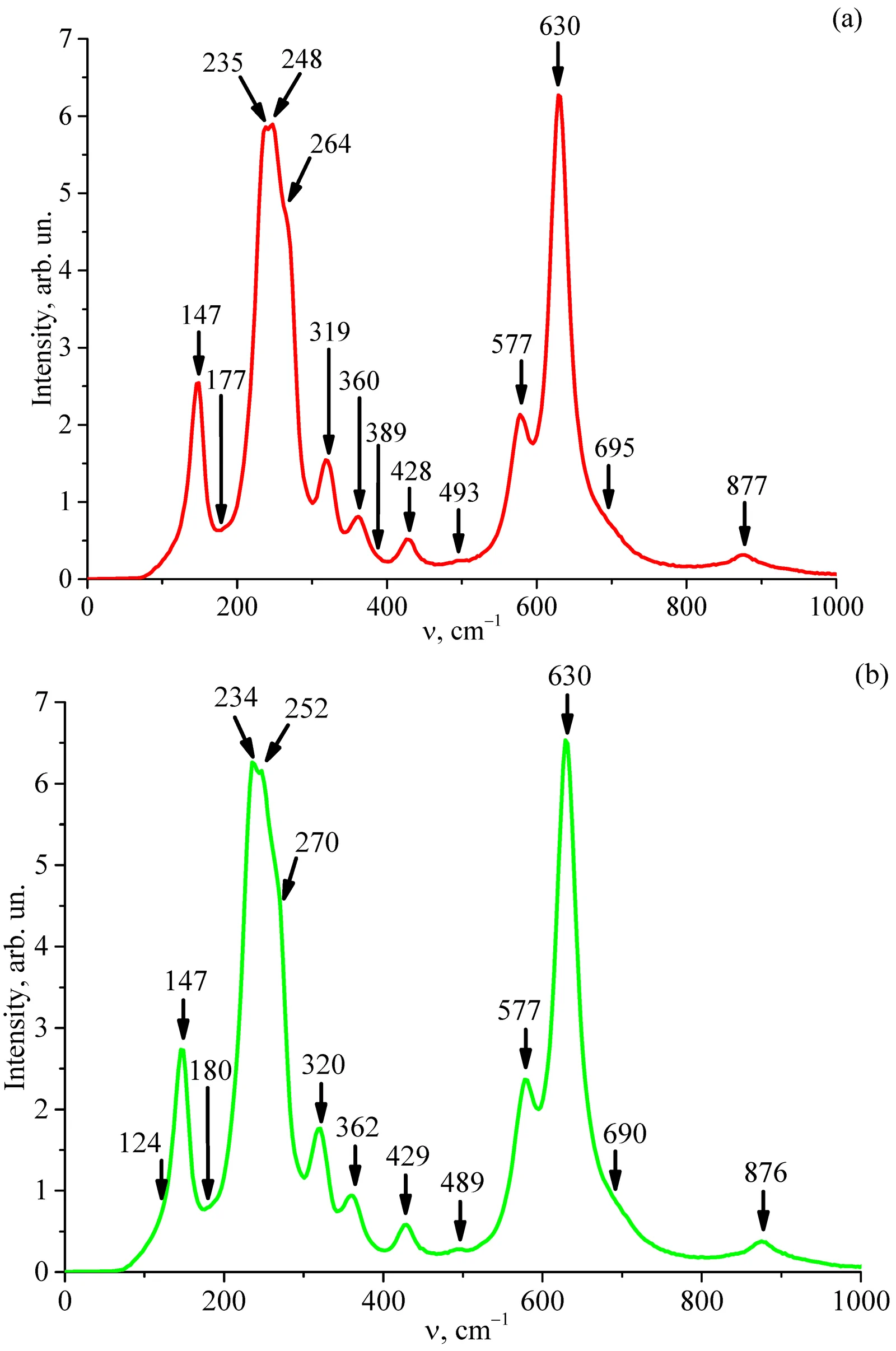
Raman spectra of LiNbO_3_:Tb^3+^(2.25 wt%) (**a**) and LiNbO_3_:Tb^3+^(2.9 wt%) (**b**) plates in the frequency range of fundamental vibrations of the crystal lattice.

The Raman spectra of LiNbO_3_:Tb^3+^(2.25 wt%) and LiNbO_3_:Tb^3+^(2.9 wt%) plates, recorded in the range of 65–1000 cm^−1^, are shown in Figure 4.

From Figure 4, it is evident that a number of lines of different intensities are observed in the recorded Raman spectra. Raman lines with frequencies of 147, 234–235, 248–252, 264, 270, 319–320, 360–362, 428–429, 577, 630, and 876–877 cm^−1^ correspond to fundamental vibrations of the A_1_(z)- and E(x,y)-symmetry types [60,61,62,63,64,65,66,67]. The lowest-frequency line of 124 cm^−1^ does not correspond to fundamental phonons, and its nature is debatable [68,69,70,71]. In these papers, this line is attributed to a biphonon—two-particle states of acoustic phonons with a total wavevector equal to zero. A weak Raman band can be seen near 180 cm^−1^. There are different interpretations for this line in the literature: in [72], it is attributed to mixed phonons, and in [60,73,74,75], to fundamental phonons 2E(x,y)TO, which, however, is not confirmed in other works [61]. In [76,77,78,79], the Raman line near 186 cm^−1^ was attributed to fundamental phonons E(x,y) for pure and doped crystals. For the Raman line near 690 cm^−1^, there are also different assignments in the literature. The authors of [73,80] believed that this line relates to two-phonon processes. In an earlier work [81], this line was attributed to fundamental phonons A_1_(z), but, according to the calculated data of [82], this line should be attributed to fundamental doubly degenerate phonons 9E(x,y)TO. It should be noted that the line at 690 cm^−1^ is most clearly manifested in the Raman spectrum of hydrogen-enriched LiNbO_3_ crystals [83]. In the Raman spectra of a congruent LiNbO_3_ crystal (R = 0.946) at temperatures above 1000 °C, a broad band with maxima near 700 cm^−1^ was recorded without any interpretation [84].

**Figure 5:**
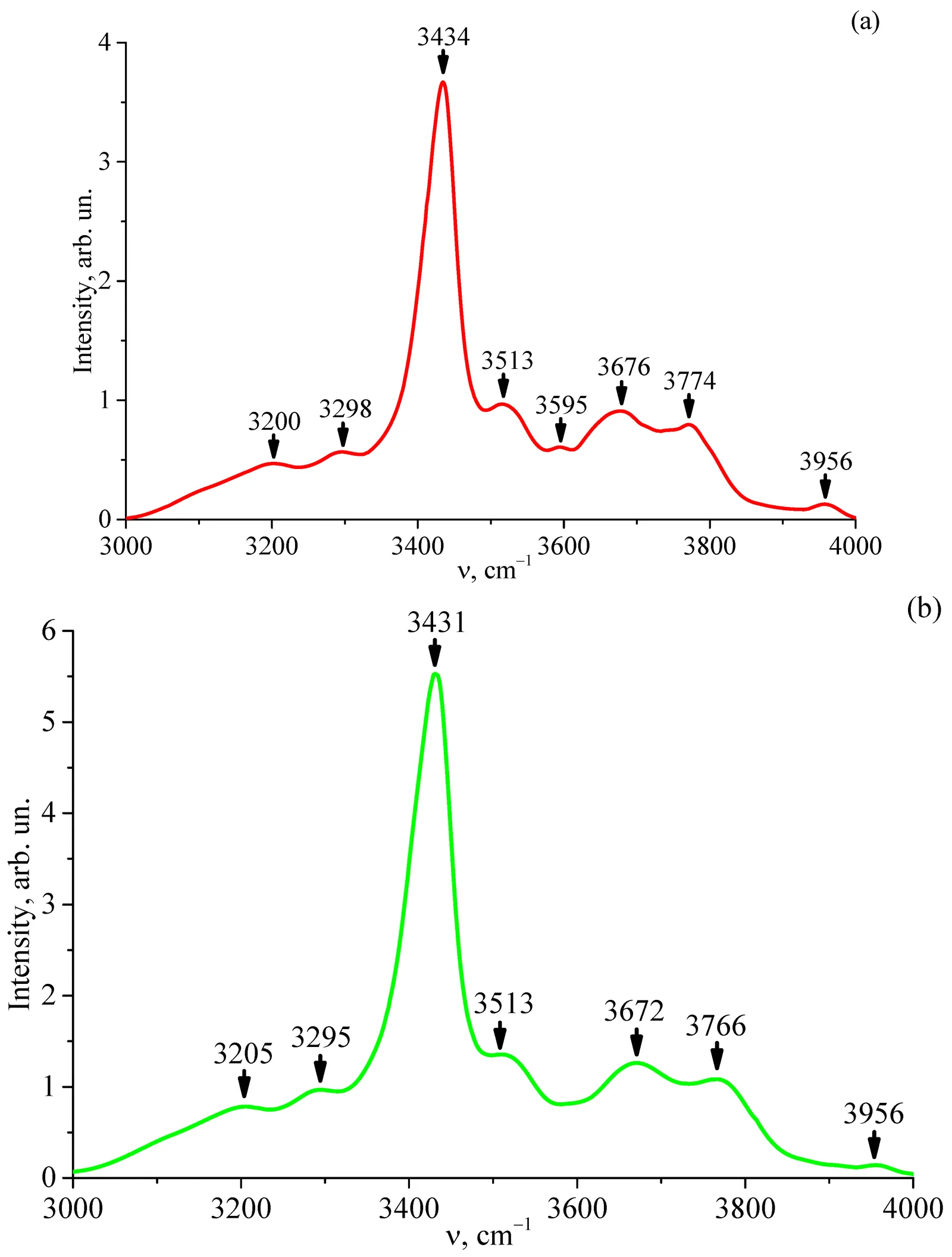
Raman spectra of LiNbO_3_:Tb^3+^(2.25 wt%) (**a**) and LiNbO_3_:Tb^3+^(2.9 wt%) (**b**) plates in the frequency range of 3000–4000 cm^−1^.

Figure 5 illustrates the Raman spectra of LiNbO_3_:Tb^3+^(2.25 wt%) and LiNbO_3_:Tb^3+^(2.9 wt%) plates in the frequency range of 3000–4000 cm^−1^, in which lines corresponding to the stretching vibrations of OH^−^-groups should appear.

The most intense line in the Raman spectrum, with a frequency of 3434 cm^−1^ in the spectrum of the LiNbO_3_:Tb^3+^(2.25 wt%) plate and with a frequency of 3431 cm^−1^ in the spectrum of the LiNbO_3_:Tb^3+^(2.9 wt%) plate, corresponds to the stretching vibrations of OH^−^-groups. Note that the spectral position of this line is shifted toward lower frequencies relative to the undoped congruent LiNbO_3_ crystal [36]. Interpretation of the remaining lines in the Raman spectrum of the LiNbO_3_:Tb^3+^(2.25 wt%) and LiNbO_3_:Tb^3+^(2.9 wt%) plates in the spectral region of 3000–4000 cm^−1^ is currently difficult.

It should be noted that the currently available results of studies using Raman spectroscopy in the region of stretching vibrations of OH^−^-groups are rather few and contradictory [28,36,85,86], and their interpretation requires significant development. IR absorption spectra can bring some clarity to the interpretation of the Raman spectra observed in the frequency range of 3000–4000 cm^−1^ of non-centrosymmetric LiNbO_3_:Tb^3+^(2.25 wt%) and LiNbO_3_:Tb^3+^(2.9 wt%) crystals. In non-centrosymmetric crystals, including the LiNbO_3_:Tb^3+^ crystal, fundamental vibrations active in the Raman spectrum are also active in the IR absorption spectrum [25]. In centrosymmetric crystals, the alternative exclusion rule operates: fundamental vibrations active in the Raman spectrum are forbidden by selection rules in the IR absorption spectrum [25]. The IR absorption spectra in the region of stretching vibrations of OH^−^-groups of LiNbO_3_ single crystals of different compositions (nominally pure of different stoichiometries and doped with a wide range of different metals) have been studied in great detail in the literature and, in many cases, reliably interpreted [18,19,32,33,34,35,36].

### 3.4. IR Absorption Spectra

**Figure 6:**
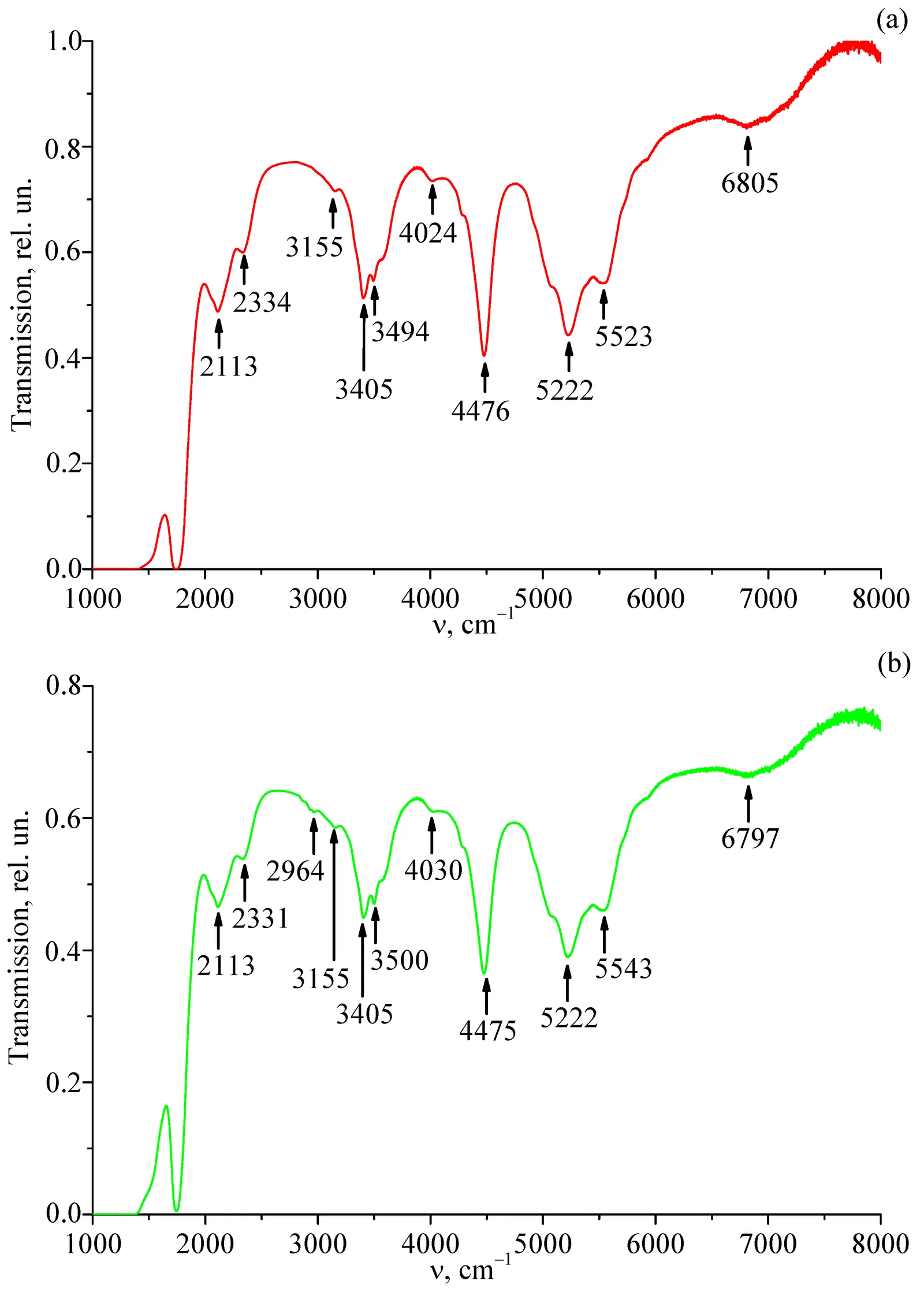
IR absorption spectra of LiNbO_3_:Tb^3+^(2.25 wt%) (**a**) and LiNbO_3_:Tb^3+^(2.9 wt%) (**b**) plates in the frequency range of 1000–8000 cm^−1^.

Figure 6 shows the IR absorption spectra recorded in a wide frequency range (1000–8000 cm^−1^) of single-crystal plates of LiNbO_3_:Tb^3+^(2.25 wt%) and LiNbO_3_:Tb^3+^(2.9 wt%).

From Figure 6, it is evident that several lines are recorded in the IR absorption spectra, which can be divided into groups separated by an energy gap for ease of analysis. The first group includes two lines at 2113 and 2329–2334 cm^−1^, which can be attributed to the absorption bands of the CO and CO_2_ groups. Previously, these absorption lines were also observed in the IR absorption spectra of some doped LiNbO_3_ and LiTaO_3_ single crystals in the frequency range of 2100–2400 cm^−1^ [86,87].

It should be noted that the absorption line with a frequency of 2964 cm^−1^ appears only in the IR absorption spectrum of the LiNbO_3_:Tb^3+^(2.9 wt%) plate and is absent in the IR absorption spectrum of the LiNbO_3_:Tb^3+^(2.25 wt%) plate. A low-intensity absorption line with a frequency of ≈3155 cm^−1^ was previously recorded in the IR absorption spectrum of LiNbO_3_:Tb^3+^ crystals with under-threshold concentrations of terbium ions LiNbO_3_:Tb^3+^ (0.48 and 2.21 wt%) [21]. However, the assignment of lines with frequencies of 2964 and 3155 cm^−1^ in the literature has not been performed to date.

The second group of lines in the IR absorption spectrum of the LiNbO_3_:Tb^3+^(2.25 wt%) and LiNbO_3_:Tb^3+^(2.9 wt%) plates is located in the range of 3000–4000 cm^−1^. In this spectral range, the manifestation of stretching vibrations of OH^−^-groups is expected. In the IR absorption spectrum of the pure congruent LiNbO_3_ crystal in this frequency range, there are three lines with frequencies of 3470, 3483, and 3486 cm^−1^, corresponding to the stretching vibrations of OH^−^-groups [19,32]. In the IR absorption spectra of LiNbO_3_:Tb^3+^(0.1, 0.48, and 2.21 wt%) [21] crystals with an under-threshold terbium concentration, these three lines merge into a broad line with a maximum at 3487 cm^−1^, located against the background of broad and intense lines with frequencies of 3437 and 3569 cm^−1^. In this case, the frequency of the maximum at 3487 cm^−1^ increases to 3493 cm^−1^, and the intensity of the maximum decreases significantly as the crystal composition approaches the concentration threshold at 2.2–2.3 wt% Tb^3+^ [21]. In the IR absorption spectrum of the after-threshold LiNbO_3_:Tb^3+^(2.25 wt%) and LiNbO_3_:Tb^3+^(2.9 wt%) plates, the stretching vibrations of OH^−^-groups correspond to a broad line with a frequency of 3494–3498 cm^−1^ (Figure 6). The observed increase in the frequency of the stretching vibrations of OH^−^-groups with increasing terbium concentration in the LiNbO_3_:Tb^3+^ crystal indicates an increase in the quasi-elastic constant of the O-H bond. A similar shift in the frequency of stretching vibrations of OH^−^-groups with increasing magnesium and zinc concentrations was observed in the IR absorption spectrum of LiNbO_3_:Mg and LiNbO_3_:Zn single crystals with changes in the localization features of magnesium and zinc ions in the crystal [88,89,90].

**Figure 7:**
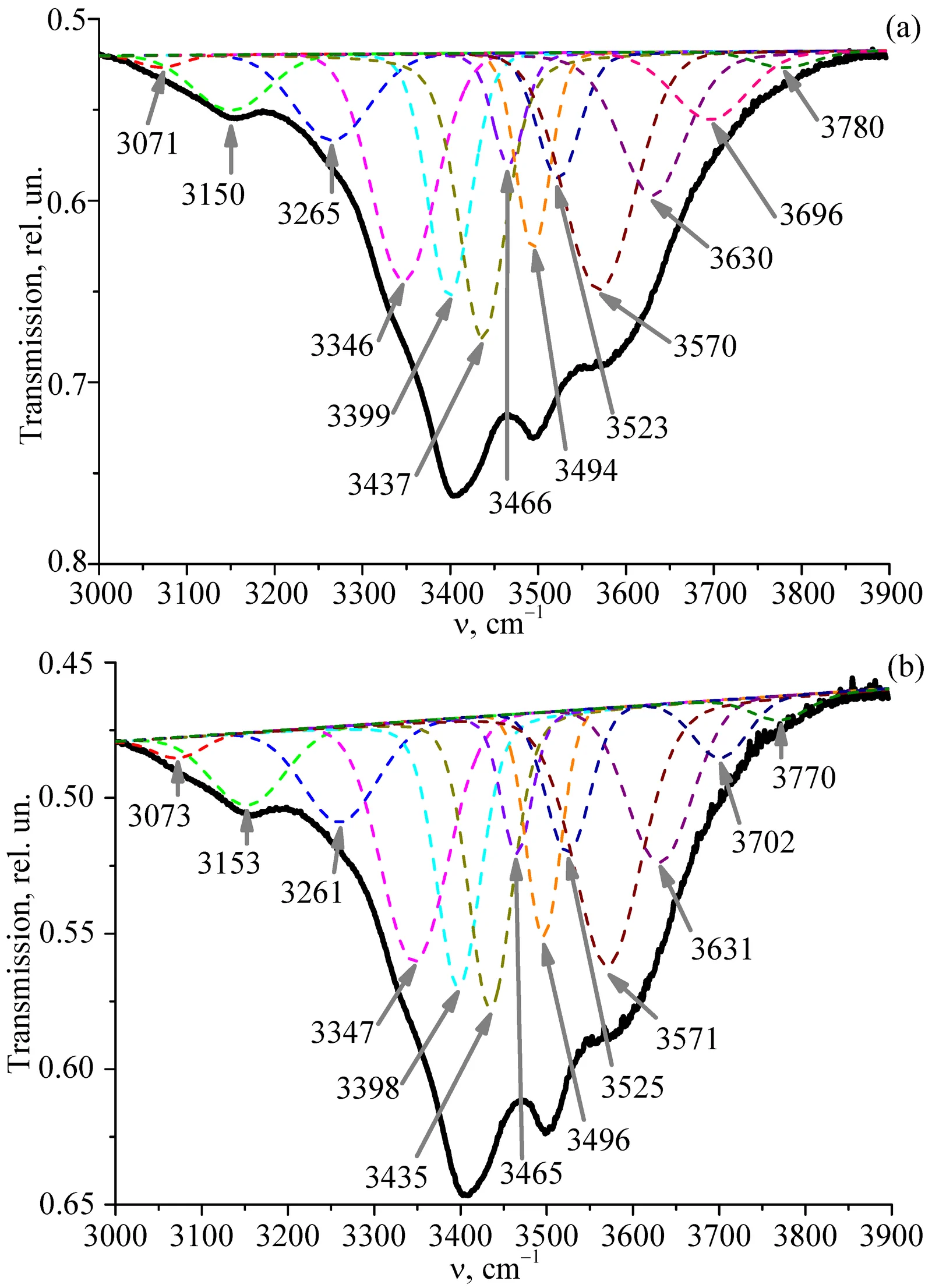
IR absorption spectra of LiNbO_3_:Tb^3+^(2.25 wt%) (**a**) and LiNbO_3_:Tb^3+^(2.9 wt%) (**b**) plates in the frequency range of 3000–4000 cm^−1^.

Figure 7 shows, in more detail, a fragment of the IR absorption spectrum in the region of 3000–4000 cm^−1^ of the after-threshold LiNbO_3_:Tb^3+^(2.25 wt%) and LiNbO_3_:Tb^3+^(2.9 wt%) plates. From this figure, one can see the multicomponent structure of the recorded spectra. Table 1 presents the main parameters of the spectral lines obtained after decomposing the multicomponent spectrum observed in the frequency range of 3000–4000 cm^−1^ into components and also gives the volume concentration of the OH^−^-groups in the crystals, calculated by us using the Klauer method [91]. The multicomponent appearance of the IR absorption spectrum in the frequency range of 3000–4000 cm^−1^ may be due to the fact that, according to the data of work [10], in the IR absorption spectrum of the LiTaO_3_:Tb^3+^ crystal (and, obviously, the LiNbO_3_:Tb^3+^ crystal), the manifestation of electronic f-f transitions is possible, which is forbidden by the selection rules for a free Tb^3+^ ion, but, in some cases, for example, under the influence of the features of the crystal field on the Tb^3+^ ion, this prohibition can be lifted. The lines corresponding to such f-f transitions can significantly overlap with the lines corresponding to the stretching vibrations of OH^−^-groups.

The shape, location, and number of lines in the IR absorption spectrum of various LiNbO_3_ crystals, corresponding to the stretching vibrations of OH^−^-groups, depend on the stoichiometry (R value) and the amount of the dopant in the crystal [18,19,32,36,87,88,89,90]. In the IR absorption spectrum of a strictly stoichiometric LiNbO_3_ crystal (R = 1), in which the shape of the O_6_ oxygen octahedra is close to the shape of an ideal octahedron, there is only one narrow line with a frequency of 3466 cm^−1^ [18]. In the IR absorption spectrum of non-stoichiometric (R ≠ 1) LiNbO_3_ crystals (pure and doped), in addition to the line with a frequency of 3466 cm^−1^, there are several more lines [19,32,36,87,88,89,90]. In this case, the number of lines is determined by the features of the deviation in the shape of the oxygen octahedra O_6_ of non-stoichiometric crystals from the shape of an ideal octahedron.

This circumstance makes it possible to group the absorption lines in the 3000–4000 cm^−1^ range into several subgroups. The first subgroup of lines with frequencies in the range of 3460–3500 cm^−1^ is linked to the violation of stoichiometry in the crystal due to the formation of point defects (V_Li_^−^) and (Nb_Li_^4+^) and the resulting increase in the quasi-elastic constant of the O-H bond. As a result, the frequency of the line corresponding to the stretching vibrations of OH^−^-groups increases. Indeed, it is evident from Figure 5 that in the IR absorption spectrum of the after-threshold LiNbO_3_:Tb^3+^(2.25 wt%) and LiNbO_3_:Tb^3+^(2.9 wt%) plates, there is an absorption line with a frequency of ≈3466 cm^−1^ and an absorption line with a higher frequency of ≈3495 cm^−1^. The frequency of this line is determined by the quasi-elastic constant of the O-H bond (determined by the value of R), which is close to unity, and by the formation of a complex defect (V_Li_^−^)-OH in the crystal structure. The intensity of the absorption line with a frequency of ≈3495 cm^−1^ in the spectrum of the LiNbO_3_:Tb^3+^(2.9 wt%) crystal is somewhat lower, as is the value of R (Table 1).

The second subgroup in the IR absorption spectra of the LiNbO_3_:Tb^3+^(2.25 wt%) and LiNbO_3_:Tb^3+^(2.9 wt%) plates includes absorption lines with frequencies of 3491–3600 cm^−1^, the presence of which is due to a change in the features (mechanism) of the incorporation of the Tb^3+^ dopant into the structure of the LiNbO_3_ crystal. According to the data in [29], the IR absorption spectrum of the double-doped LiNbO_3_:Mn:Tb crystal contains an absorption line of 3492.4 cm^−1^. In our case, the IR absorption spectrum of the after-threshold LiNbO_3_:Tb^3+^(2.25 wt%) and LiNbO_3_:Tb^3+^(2.9 wt%) plates contains a line ≈3494 cm^−1^. This spectral component, according to the data of work [91], can be attributed to the stretching vibrations of hydrogen atoms in the complex defect 3(Tb_Li_^2+^)-(V_Nb_^5−^)-OH^−^ or in the complex defect (Tb_Li_^2+^)-2(V_Li_^−^)-OH^−^. The dopant Tb^3+^ in the LiNbO_3_:Tb^3+^(2.25 wt%) crystal occupies the main positions of the Li^+^ ion. Also, in this crystal, there is a larger number of point defect centers (V_Nb_^5−^) compared to the LiNbO_3_:Tb^3+^(2.9 wt%) crystal [26].

Thus, we assume the formation of complex defects of the type 3(Tb_Li_^2+^)-(V_Nb_^5−^)-OH^−^ in the structure of the after-threshold LiNbO_3_:Tb^3+^ plates, which correspond to an absorption band with a frequency of ≈3494 cm^−1^. According to the data in work [26], with an increase in the concentration of terbium ions in the LiNbO_3_ crystal, the number of defect centers Tb_Li_ decreases (a terbium ion in the position of a lithium ion in the ideal structure) due to the fact that terbium begins to occupy vacant octahedra. This causes a local deformation of the crystal lattice, which is manifested in the IR absorption spectrum of the LiNbO_3_:Tb^3+^(2.9 wt%) plate in the shift of the absorption line corresponding to the stretching vibration of hydrogen atoms in the complex defect 3(Tb_Li_^2+^)-(V_Nb_^5−^)-OH^−^ to 3500 cm^−1^ (Figure 4, Table 1).

Using the Klauer method [92] and the spectra shown in Figure 7, we calculated the volume concentration of OH^−^-groups in the LiNbO_3_:Tb^3+^(2.25 wt%) and LiNbO_3_:Tb^3+^(2.9 wt%) plates (see Table 1). Because the studied single-crystal plates had different thicknesses (L = 2.27 and 1.55 mm), the results of calculating the concentration of OH^−^-groups were normalized to the thickness of the plates. According to the calculation, the maximum value of the concentration of OH^−^-groups is characteristic of the LiNbO_3_:Tb^3+^(2.9 wt%) plate, even taking into account the high value of the R, due to the formation of complex defects: (V_Li_^−^)-OH (according to estimates, their number is very small) and 3(Tb_Li_^2+^)-(V_Nb_^5−^)-OH^−^. The obtained data are consistent with the results of the microstructure study and optical absorption spectra.

The third group of lines recorded in the IR absorption spectra is located in the frequency range >4000 cm^−1^. The five lines in this group may relate to composite tones (for example, stretching + libration vibrations of OH^−^-groups) or to transitions in the terbium ion. For example, the lines at 4475, 5222, and 5523 (5543) cm^−1^ are close to the literature data on the ^7^F_6_ → ^7^F_3_, ^7^F_6_ → ^7^F_2_, and ^7^F_6_ → ^7^F_1_ transitions of the terbium ion, respectively, in various matrices [93,94,95,96,97].

Finally, the following should be noted. A comparison of Figure 5, Figure 6 and Figure 7 reveals that the frequencies of the lines corresponding to OH^−^-group stretching vibrations in the Raman and IR absorption spectra differ markedly, which is unusual. In non-centrosymmetric crystals, according to the selection rules for fundamental lattice vibrations, the fundamental vibrations active in the Raman spectrum are also active in the IR absorption spectrum [25]. The difference in the frequencies of the lines under discussion can be explained by the fact that OH^−^-groups in the LiNbO_3_ crystal structure are defects; therefore, their stretching vibrations do not correspond to fundamental lattice vibrations. When describing the symmetry properties of crystals using group theory, defects in the crystal structure are ignored, and the crystal is considered a homogeneous, anisotropic, defect-free medium. It should also be noted that there are currently no theories that allow for a sufficiently accurate determination of the vibrational activity in the spectra of highly defective crystals.

## 4. Conclusions

The peculiarities of the defect structure of after-threshold single-crystal plates of LiNbO_3_:Tb^3+^(2.25 wt%) and LiNbO_3_:Tb^3+^(2.9 wt%), which are proposed as optical materials for converting laser radiation, were studied using: optical microscopy, optical absorption spectra, Raman spectra in a wide frequency range, including the frequency range of 100–1000 cm^−1^, corresponding to fundamental vibrations of the crystal lattice, and the frequency range of 3000–4000 cm^−1^, corresponding to the stretching vibrations of OH^−^-groups; and the IR absorption spectra in the frequency range of 1000–8000 cm^−1^.

The macro- and microstructure of LiNbO_3_:Tb crystals with Tb concentrations of 2.25 and 2.9 wt% was studied using optical microscopy. It was found that even a slight increase in terbium content (less than 1 wt%) leads to a dramatic evolution of the growth structure: the crystal with 2.25 wt% exhibits distinct concentric growth domain rings, while, at 2.9 wt%, these rings begin to merge and a continuous etching field of the negative domain is observed in places. Self-organization elements in the form of fractal structures reminiscent of Sierpinski triangles were detected in both compositions, with such structures occupying a significantly larger volume of the crystal at 2.9 wt%.

It was found that for the LiNbO_3_:Tb^3+^(2.25 wt%) plate, a smaller shift of the fundamental absorption edge toward shorter wavelengths (λ_20_ = 385.55 nm) was observed compared to that observed for the plate with LiNbO_3_:Tb^3+^(2.9 wt%) (λ_20_ = 387.1 nm). This fact indicates a higher compositional homogeneity of the LiNbO_3_:Tb^3+^(2.25 wt%) plate (due to a lower concentration of Nb_Li_ point defects in it and a higher stoichiometry) compared to the LiNbO_3_:Tb^3+^(2.9 wt%) plate. It is established that the Raman spectra of the LiNbO_3_:Tb^3+^(2.25 wt%) and LiNbO_3_:Tb^3+^(2.9 wt%) plates coincide quite well, both in the region of frequencies of fundamental vibrations of the crystal lattice and in the region of stretching vibrations of OH^−^-groups. Minor differences in the vibration frequencies are explained by differences in the values of the quasi-elastic constants. However, in the range of stretching vibrations of OH^−^-groups, the frequencies of the lines in the Raman spectra and the lines in the IR absorption spectra of the after-threshold LiNbO_3_:Tb^3+^(2.25 wt%) and LiNbO_3_:Tb^3+^(2.9 wt%) plates differ significantly. This can only happen for fundamental lattice vibrations if the crystal has a center of symmetry. However, LiNbO_3_:Tb^3+^ crystals do not have a center of symmetry. We explain the experimental fact of the frequency discrepancy by the fact that the OH^−^-groups in the LiNbO_3_ crystal structure are defects, and, therefore, the stretching vibrations of OH^−^-groups are not fundamental lattice vibrations. The existing selection rules for fundamental lattice vibrations, developed on the basis of group theory, do not apply to the defect sublattice.

## Figures and Tables

**Table 1:** Values of frequencies (ν, cm^−1^), intensities (I, arb. units), and widths (S, cm^−1^) of lines in the IR absorption spectra; the fundamental absorption edge (nm); and the concentration of OH^−^-groups (C (OH^−^-)/cm^−3^) in after-threshold LiNbO_3_:Tb^3+^ plates.

Plate	ν, cm^−1^	I	S, cm^−1^	C(OH^−^), cm^–3^	Absorption Edge, nm	R = Li/Nb
LiNbO_3_:Tb^3+^ (2.25 wt%)	3436	0.15	63.15	1.72·10^18^	385.55	1.005
	3466	0.05	42.43			
	3494	0.10	45.52			
	3523	0.06	57.14			
	3569	0.12	94.48			
LiNbO_3_:Tb^3+^ (2.9 wt%)	3434	0.10	60.37	2.16·10^18^	387.1	0.965
	3465	0.04	42.75			
	3496	0.08	46.85			
	3524	0.05	59.88			
	3570	0.09	92.40			

## Data Availability

The raw data required to reproduce these findings are available from corresponding author A.Yu.P. upon reasonable request.
